# Assessment of the status quo of social responsibility performance of inclusive kindergartens: Evidence from China

**DOI:** 10.1371/journal.pone.0272742

**Published:** 2022-11-28

**Authors:** Yang Lv, Min Wu, Chenwei Ma, Xinxin Hao, Xun Zeng

**Affiliations:** 1 College of Teachers, Chengdu University, Chengdu, China; 2 School of Public Administration, Sichuan University, Chengdu, China; 3 School of Management, Sichuan University of Science and Engineering, Zigong, China; Neijiang Normal University, CHINA

## Abstract

China is determined to accomplish universal preschool education by asking the kindergartens to participate in social responsibility programs. This study intends to assess the level of participation of inclusive kindergartens in social responsibility programs. This study uses the Delphi expert method, integrated ISO26000 International Standard Guidelines for Social Responsibility, CSR (Corporate Social Responsibility) Scale, and the characteristics of the preschool education industry to construct a social responsibility evaluation model for inclusive kindergartens. It includes five dimensions (responsibility management, customer responsibility, employee responsibility, social service, and organizational responsibility) to show the social responsibility status of kindergartens. Data was collected from 832 respondents from 27 provinces, cities, and regions in China. This study reveals that the overall performance of social responsibility of inclusive kindergarten (3.67) is better, while organization responsibility (3.91) shows the highest performance. In comparison, customer (3.63) and staff responsibility (3.63) deliver average performance, and responsibility management (3.56) offers lower performance. The statistical analysis shows that the nature of kindergartens, whether inclusive or not, the number of classes, years of establishment, the distribution area, and performance are different. Kindergartens should have certain social values, including specific behaviors and participating in social activities in the spirit of social service. They should ensure preschool teacher’s professional and vocational development through multiple subjects’ synergetic governance. In addition to fulfilling the teachers’ social responsibility and professional development, the findings can put forward the cooperation with the government, social organizations, and kindergartens to improve teachers’ professional quality and social responsibility.

## 1. Introduction

In recent years, preschool education has received significant attention from the Chinese government. The Central Committee of the Communist Party of China and the State Council clearly emphasized on China’s education inclusiveness by 2020 in the ‘Several Opinions on the Deepening Reform and Standard Development of Preschool Education in 2018’. The coverage rate of kindergartens (hereafter referred to as ‘*Puhui* kindergartens’) will reach 80% by 2035 [1]. It is a public service system for preschool education that reasonably covers both urban and rural areas.

*Puhui* is the pinyin expression of 普惠. *Puhui (普惠)* is derived from Chinese *Pu* (普), indicating universality, and *Hui* (惠) means a benefit. It refers to something reasonably excellent and inexpensive. In ancient Chinese, *hui* means benevolence, implying a sense of affection for and service to others. Currently, *hui* refers to equitable distribution of benefits and something of high quality. Thus, *Puhui* implies that early childhood education should be a universal, inexpensive, accessible, and accountable program available to all low-income families.

Moreover, there has been no agreement on which kindergartens in China should be designated *Puhui*. When the reform and opening-up program was implemented during the 1990s, the central government imposed a program known as government retreats [2]. Educational authorities have delegated funding and oversight of early childhood education (ECE) to private and non-governmental entities. As a result, public kindergartens have been forced to close, suspend, merge, transform or sell.

Before 2010, kindergartens were typically divided into three types: public kindergartens administered by educational departments, public kindergartens run by non-educational departments, and private kindergartens with a license [3]. The educational authorities and other public organizations control, fund, and regulate the first two types. They are widely believed to have higher standards of instruction, facilities, and other resources than their private counterparts [4]. Additionally, participation in public kindergarten is a more economical option for Chinese families because governments regulate public kindergarten tuition and other expenditures [5]. However, public kindergarten enrollment is restricted and skewed toward higher-income families in urban areas [6]. To address the ‘3A’ issues plaguing the preschool education system–accessibility (it’s challenging to get into a kindergarten), affordability (it’s more expensive than a university), and accountability (low quality and no monitoring mechanism), the concept of *Puhui* kindergarten is offered [2].

According to 2019 statistics by China’s Ministry of Education, the country’s kindergartens were 281174; among them, private gartens are 173,236 (61.61%), and the urban private garten accounted for 76.96%. The gross enrollment rate of preschool education in China in 2019 was 83.4%. Though the enrollment rate has been increasing over the last few years, preschool education’s unbalanced and inadequate development has not been fundamentally changed, and it is generally inefficient and unfair. The current number and coverage of kindergartens cannot support the strategic goal of public service inclusiveness in preschool education.

Tian [3] conducted a study on *Puhui* kindergarten in 2021 and reported that these kindergartens are still at the initial stage and require government support to improve education quality and engage in social responsibility mechanisms. Zhou et al. [2] analyzed the 3A challenges of *Puhui* kindergartens. These challenges are accessibility, affordability, and accountability. They reported that the affordability and accountability of the Puhui kindergartens are resolved properly, but the accessibility of the students has still a challenge. According to Zhou et al. [7], Chinese parents rated *Puhui* kindergarten’s quality well, but they rated the school’s financial allocation and educational recompense for underprivileged children poorly. The latent profile analysis found low, medium, and high-level parent rating profiles, which differed by educational background, geographic location, kindergarten type, and monthly expenses. The kindergarten type had a critical influence on *Puhui* kindergarten’s appraisal.

The ‘Deepening Reform Opinions’ also clarified the strategic positioning of universal and inclusive, safe, and high-quality preschool education development at this stage [8]. The group characteristics of preschool education audiences with an early age and a large base determine the safety and inclusiveness of preschool education institutions. So, it requires enhancing the overall awareness of social responsibility in the preschool education industry and create a positive and harmonious atmosphere of social responsibility [9]. It is also urgent to develop the social responsibility evaluation model and specific dimensions of kindergartens to understand the current situation in China. The main difference between public interest kindergartens (PIK) and private kindergartens are funding sources, expenses, and other related facilities. Private kindergartens are relatively expensive and solely dependent on students’ tuition and other fees. PIK’s primary goal is to provide high-quality, low-cost early childhood education services to the general public [10]. Factors that hinder the transformation of kindergartens to PIK are complex and multifaceted [11]. Before kindergarten transitions to PIK, conceptual changes and policy modifications have to be undertaken to promote democratization and highlight the social responsibility of ECE. While public and private kindergartens may have different development orientations, both serve the public interest [12]. The ECE system needs to prioritize service and the public good over profit to encourage the transformation of kindergartens to PIK [13]. It requires support and supervision from the government and the cooperation of the private sector.

Many studies already have focused on ECE [6,14–16], challenges of preschool systems [17–19], kindergarten care [20,21], and *Puhui* kindergartens [2,3,7] in China, but there is almost no focus on the social responsibility of *Puhui* kindergartens which it should render for society. Therefore this study addresses three research questions: (a) Are *Puhui* kindergartens participating in any social responsibility programs? (b) What is their social responsibility system participation status across China?; and (c) Is there any association among various dimensions of the social responsibility system? Since this issue is urgent to ensure equal access, affordability, and accountability in preschool education across China, this study intends to address the research gap by exploring the level of participation of inclusive kindergartens in social responsibility programs.

## 2. Theoretical framework

### 2.1 Evaluation model of the social responsibility of *Puhui* kindergarten

Scholar’s view on ‘universal inclusiveness’ of kindergartens focuses on the analysis of the connotation, current situation and characteristics of the inclusiveness of preschool education, along with reasons, measures and strategies for developing the inclusiveness of preschool education [22–26]. With the exposure of a series of negative events in kindergartens [27], scholars have conducted a series of studies on how kindergartens can ensure the provision of ‘safe and high-quality educational services [28–31]. Only a few scholars consider private kindergartens socially responsible [32].

Many scholars focus on the concept, dimensions, and evaluation of social responsibility but mainly in the field of corporate social responsibility (CSR) [33–36]. While the existing studies in China mainly focus on the corporate social responsibility assessment of listed companies [35] and state-owned enterprises. At the same time, due to the unique characteristics of China’s social culture and management systems, it requires more attention to local connotations [37]. The social responsibility of kindergartens includes universal inclusiveness [7], safe and high-quality education services [38], government financial support and timely feedback to parents [29], participation in community social services [39], and the protection of the interests of teachers and employees [40].

Therefore, this study also consults the classical theory and local characteristics for defining and selecting the scope of social responsibility of *Puhui* kindergarten, its determinants, and evaluation dimensions.

Firstly, this research determines the scope of the social responsibility assessment of *Puhui* kindergarten by drawing on and absorbing the corporate social responsibility scales [35] and Western researchers [41–44]. At present, the measurement and evaluation of corporate social responsibility in the academic circle seem to have a holistic evaluation [42], around the evaluation of stakeholders [44,45], management [46], and ‘economy, law, environment, society’ four-dimensional evaluation [46]. The main evaluation elements are environmental responsibility, responsibility management, employee responsibility, community responsibility, consumer responsibility and other elements.

Secondly, the two core standards for social responsibility measurement in the IS026000 are ‘core theme of social responsibility’ and ‘guidelines for integrating social responsibility into the whole organization’. The three aspects of social responsibility are evaluated and considered, like awareness enhancement and social responsibility capacity building, setting the organization’s social responsibility direction, and incorporating social responsibility into organizational governance, systems and procedures.

Third, the mission of Puhui Garten is to ensure quality education with reasonable fees for early childhood care and education services. Its decision-making and activities should promote the harmonious and healthy development of children’s bodies and minds, and satisfy the government, parents of children, communities, and organizations as the client [7]. Therefore, this research has preliminarily determined the scope, evaluation dimensions, and indicators of the social responsibility of the Puhui kindergarten. The major indicators are responsibility management, employee responsibilities, customer responsibility, society Service, legal responsibility, and organizational responsibility. A total of 21 specific evaluation indicators have been considered.

### 2.2 Social responsibility evaluation index

According to the screening results of the first round of indicators, the remaining 17 indicators were again considered. The evaluation dimension has been adjusted according to context. The connotation of the evaluation index of legal responsibility is similar to that of the construction of an organization’s compliance system and training. Therefore, the legal responsibility and the organization’s responsibility are merged into the organization’s responsibility. Finally, the inclusive gartens’ social responsibility evaluation index system has been developed, comprising 5 evaluation dimensions and 15 specific evaluation indicators (Table 1).

**Table 1:** Scope of social responsibility evaluation index of *Puhui* kindergarten.

Social Responsibility of Puhui Garten	Evaluation dimension	Evaluation index
	Responsibility management	Organization mission, vision, values
		Organizational social responsibility management system and implementation
		Information transparency
	Customer Responsibility	Service quality and customer safety
		Customer information protection
		Customer complaint and dispute handling
	Employee responsibility	Employee rights
		Employee Development
		Labor relations
	Social service	Community participation and development
		Public welfare behavior
		Inclusiveness and equal opportunity
	Organizational responsibility	Compliance system construction and training
		fair competition
		Green environment and management

In the social responsibility evaluation index, a total of 18 measurement indicators have been identified, of which responsibility management includes 3 topics [21], 6 questions about customer responsibility [47], 3 indicators of employee responsibility [48], 3 indicators in social services [49], 3 indicators of organizational responsibility [50]. The variables were measured by 5 points Likert scale. The higher the score, the better the performance of this indicator. To further simplify the information and provide a good degree of distinction, those with a score of 3 or less are regarded as average performance, and those with a score of 3 or more are considered good performance.

## 3. Methodology

### 3.1 Data sources and collection

The data collection survey was carried out in various provinces, cities and regions of China. Through online and offline questionnaire surveys, 1,536 questionnaires were distributed to 400 preschool education institutions in 31 provinces, municipalities, and autonomous regions in China. A total of 1,050 questionnaires were collected. Finally, 832 valid questionnaires are included in the study after excluding invalid and incomplete questionnaires. The results show that the Cronbach coefficient of the overall questionnaire data is 0.928, and the internal consistency of the questionnaire data is good. The sample covers 27 provinces, 103 cities, and 261 preschool education institutions.

### 3.2 Indicator selection and approaches for measurement

The Delphi expert consultation method was used to design the expert consultation questionnaire for the social responsibility evaluation indicators of *Puhui* kindergarten. Experts’ attitudes towards specific evaluation indicators were obtained, then selected the more important indicators for sorting. Summarization, statistics, and anonymous feedback to the experts were considered. The feedback was also received until preparing a consistent evaluation index of the social responsibility of the *Puhui* kindergarten. Based on experts’ consultation, this study selected 11 staff from the education administration department, 13 scholars engaged in preschool education research, and 9 doctoral and master students for the research group. In terms of questionnaire design, a five-point Likert scale like ‘very unimportant, unimportant, general, important, and very important’ is designed according to each item’s importance and scored ‘1, 2, 3, 4, and 5’ respectively. The screening criteria mainly considered the mean and standard deviation. The mean reflects the importance of the index as a whole, and the standard deviation reflects the consistency and stability of expert opinions. Because of this, the study decided to set the critical value at 3.75 points between ‘average’ and ‘important’ (considering from the quartile, it is more reasonable to select the 75th percentile after the mean is ranked from small to large) and standard deviation. The index greater than 1 is used as the criterion for index screening.

The descriptive analysis results are shown in Table 1. Among the 21 initial indicators, 4 indicators have a mean value less than 4 and a standard deviation greater than 1, including sustainable development (3.67, 1.15), anti-corruption (3.56, 1.12), and employee recruitment (3.65, 1.23), and values (3.74, 1.02). Therefore, 17 indicators were retained through the first round of screening (Table 2).

**Table 2:** Screening of social responsibility evaluation indexes of *Puhui* kindergartens.

Social Responsibility of *Puhui* kindergarten	Evaluation dimension	Evaluation index	Mean	Standard deviation
	Responsibility management	Sustainable development	3.67	1.15
		Information transparency	4.32	0.58
		Anti-corruption	3.56	1.12
		Organization mission, vision, values	4.36	0.85
		Organizational social responsibility management system and implementation	4.46	0.67
	Employee responsibility	Employee rights	4.63	0.73
		Employee Development	4.02	0.55
		Hiring	3.65	1.23
		Labor relations	4.17	0.66
	Customer Responsibility	Service quality and customer safety	4.45	0.48
		Customer information protection	4.21	0.81
		Customer complaint and dispute handling	4.58	0.74
	Social service	Values	3.74	1.02
		Community participation and development	4.56	0.69
		Public welfare behavior	4.47	0.62
		Inclusiveness and equal opportunity	4.41	0.86
	Legal liability	Compliance	4.87	0.49
		Tax payment with integrity	4.04	0.77
	Organizational responsibility	Compliance system construction and training	4.29	0.71
		fair competition	4.19	0.55
		Green environment and management	3.89	0.66

### 3.3 Ethical standard

The ethical guidelines were followed during the research process. The study was approved by the ethical review committee of Sichuan University, China. Besides, the aim and confidentiality had been explained to the interviewee and prior verbal consent was obtained from the respondent before each interview following the approval of the committee.

## 4. Results and discussions

### 4.1 Demographic characteristics of the respondents

This study has been selected some key characteristics of the respondents which are relevant to the study. The salient characteristics of the respondents have been presented in Table 3. This study explores that most of the respondents are women and between 25–35 years old. The majority are undergraduates, and their working experience is concentrated in 5–10 years. The number of employees above the management level accounts for more than half. The kindergarten age is mostly 5 years old, and the number of classes is mostly more than 5. The sample numbers of public and private kindergartens are almost equal. Among them, the generalized kindergarten is the majority, and more samples are from the eastern and coastal areas. The above information shows that the development of preschool education institutions in our country is in a stage of vigorous growth (Table 3).

**Table 3:** Demographic characteristics of the respondents.

Demographic variables	Group	Frequency	Percentage
Gender	Male	34	4.1%
	Female	798	95.9%
Age	Under 25	8	1.0%
	25–35 years old	512	61.4%
	35–45 years old	260	31.3%
	45–55 years old	52	6.3%
	Over 55 years old	0	0.0%
Education	Junior high school and below	0	0.0%
	High school / college / technical school	10	1.2%
	College	278	33.4%
	Undergraduate	536	64.4%
	Master degree and above	8	1.0%
Length of service	Less than 5 years	104	12.5%
	5–10 years	444	53.4%
	10–15 years	200	24.0%
	More than 15 years	84	10.1%
Job level	Senior management	20	2.4%
	Middle management	178	21.4%
	Grassroots management	250	30%
	General Staff	384	46.2%
Years of establishment (Number of gartens)	Within 1 year	1	0.4%
	1–5 years	23	8.8%
	5–10 years	108	41.4%
	More than 10 years	129	49.4%
Number of classes (Number of gartens)	5 or less	6	2.3%
	5–10 months	101	38.7%
	10 or more	154	59.0%
Nature (Number of gartens)	Public kindergarten	135	51.7%
	Private kindergarten	126	48.3%
Is it inclusive? (Number of gartens)	Yes	228	87.3%
	no	33	12.7%
Area (Number of gartens)	Eastern and coastal areas	124	47.5%
	Western Region	49	18.7%
	Central and northern regions	88	33.8%

### 4.2 Status of the fulfillment of the social responsibilities of Puhui Garten

The current overall level of social responsibility of inclusive gartens is above average (M = 3.67), indicating that most of the educational work of inclusive gartens. This study reveals the following characteristics of the social responsibility of the kindergarten.

First of all, the social responsibility of Puhui Garten is better performed and demonstrated when it involves the scope of implementation stipulated and guided by laws and regulations such as mandatory social responsibility. For example, 97.5% of the respondents said that their Puhui Garten had a good performance in the organization responsibility category of compliance with national laws and regulations / legal business’ (M = 4.42). This is the only item whose average value is higher than 4 points. In addition, 88.2% of the respondents said that their Puhui Garten complied with the employee rights (salary, welfare, social security, etc.) stipulated by laws, regulations, or collective agreements (M = 3.75), i.e., both are worthy of the second item. This result also verifies that the current fulfillment of the social responsibilities of inclusive gartens in China is more driven by the ‘external drive’ force of regulatory pressure from the monitoring and supervision of the relevant policies of the national government [51].

Secondly, Puhui’s social responsibility performance scores are relatively low when it involves the cognitive category of mobilization, motivation, and promotion under voluntary social responsibility and other valuable principles. For example, 42% of the interviewees said that their Puhui Garten had an average performance in the customer responsibility category of effective student and parent tracking services and management complaint system (M = 3.43). This item has the lowest score. In addition, 46.7% of respondents indicated that their Puhui Garten had an average performance in the responsibility management category of ‘regularly publicizing social responsibility reports’ (M = 3.52), and in the ‘effective use of government policy loans and subsidies’ (M = 3.52). In the two aspects of ‘providing fair career development opportunities’ (M = 3.52), 44.2% and 39% of the respondents stated that their inclusive gartens performed averagely. They were all three items worthy of the lowest score. Nearly half of the respondents think the performance is average. This result shows that the current preschool education inclusive service reform in China is still preliminary. The ‘poor admission’ situation has not been fundamentally changed. The inclusive garten lacks the ‘internal drive’ power and consciousness of social responsibility.

Third, although the overall performance of the social responsibility of Inclusive Gartens in China is better, the standard deviation of each item ranges from 1.04 to 1.38, which are all greater than 1, indicating that the degree of data dispersion is relatively large. The level of responsibility is uneven. This result shows the current unbalanced and inadequate development of preschool education in China, showing the imbalance between the public welfare nature of the inclusive garten and the capital pursuit of profit [52]. In addition, the standard deviation of the item ‘has high teaching quality and parent satisfaction’ ranks the highest with 1.38. This result shows that the current educational service quality and parent satisfaction of the inclusive gartens in China are two different levels. The division is large, and it may be due to no effective feedback system for evaluating social responsibility inside and outside kindergartens (Table 4).

**Table 4:** Status of the fulfillment of social responsibilities of Puhui Garten.

Dimension	Item	Mean	Standard deviation	Average performance (score 3 and below)		Good performance (score 3 or higher)	
				Frequency	percentage	Frequency	%
Responsibility management 3.56	There is a clear special strategic plan for social responsibility	3.62	1.10	270	37.1%	458	62.9%
	Regularly publish social responsibility reports	3.52	1.23	340	46.7%	388	53.3%
	Effective use of government policy loans and subsidies	3.55	1.12	322	44.2%	406	55.8%
Customer responsibility 3.63	The kindergarten has a comprehensive response mechanism for children’s safety accidents	3.57	1.18	266	36.5%	462	63.5%
	There is a comprehensive video surveillance system in the garten	3.73	1.21	174	23.9%	554	76.1%
	Have a unique teaching concept and detailed education and teaching plan	3.63	1.11	274	37.6%	454	62.4%
	Have high teaching quality and parent satisfaction	3.66	1.38	223	30.6%	505	69.4%
	Have parent and student personal information security management mechanism	3.75	1.20	198	27.1%	530	72.9%
	Has an effective student and parent tracking service and management complaint system	3.43	1.08	306	42.0%	422	58%
Staff responsibility 3.63	Comply with employee rights and interests (salary, welfare, social security, etc.) stipulated by laws, regulations or collective agreements	3.75	1.12	86	11.8%	642	88.2%
	Can provide fair career development opportunities	3.55	1.08	288	39.0%	440	61.0%
	Educate and train employees	3.60	1.16	246	33.7%	482	66.3%
Society service 3.65	Able to provide services for special needs (such as disabled children)	3.74	1.04	96	13.1%	732	86.9%
	Actively organize and promote social service activities in the community	3.57	1.14	196	26.9%	532	73.1%
	Have a fair and inclusive charging policy	3.64	1.15	106	14.5%	622	85.5%
Organization responsibility 3.91	Comply with national laws and regulations/legal business	4.42	1.15	18	2.4%	710	97.5%
	Have a good reputation and a positive image	3.63	1.13	145	19.9%	583	80.1%
	Safety and quality assurance of drinking water, sanitation, teaching facilities, garten buildings, etc.	3.68	1.17	148	20.3%	580	79.7%
Social responsibility		3.67					

### 4.3 Differences in social responsibility performance of various kindergartens

The values of F-test indicate that there are significant differences among kindergartens. It shows that the status quo of the performance of social responsibility of kindergartens is affected by organizational characteristics. The F- test values also show that the number of classes, private nature, inclusive nature, and social responsibility performance of kindergartens in the eastern coastal areas are 3.749, 7.390, 5.737, 9.500, and 3.970, respectively. The differences between groups were discussed according to the five dimensions of social responsibility (Table 5).

**Table 5:** Differences in social responsibility performance of various kindergartens.

Variables		Responsibility management (M±SD)	Customer Responsibility (M±SD)	Employee responsibility (M±SD)	Social service (M±SD)	Organizational responsibility (M±SD)	Social responsibility (M±SD)
Established Years	Within 1 year	4.00±0.47	3.66±1.17	3.63±0.23	3.50±0.70	3.43±1.41	3.64±0.86
	1–5 years	3.37±1.09	3.10±0.94	3.26±0.86	3.32±0.86	3.73±0.99	3.35±0.66
	5–10 years	3.55±0.99	3.65±0.86	3.59±0.92	3.63±0.90	4.01±1.04	3.69±0.71
	10 years Above	3.63±0.98	3.72±0.94	3.66±0.98	3.68±0.96	4.21±1.00	3.78±0.77
F		0.896	4.812**	2.805*	2.493	2.955*	3.749*
Class quantity	5 or less	3.03±0.90	2.61±0.86	2.93±1.14	2.73±0.75	3.31±0.83	2.92±0.68
	5–10	3.51±0.98	3.59±0.87	3.50±0.96	3.54±0.87	3.59±1.02	3.55±0.69
	10 Above	3.64±0.99	3.70±0.93	3.69±0.97	3.70±0.95	3.75±1.02	3.69±0.77
F		2.482	7.151**	4.321*	6.261**	3.396*	7.390***
kindergarten nature	Public	3.46±0.98	3.45±0.93	3.44±0.968	3.51±0.91	3.78±1.00	3.53±0.74
	Private	3.70±0.99	3.83±0.87	3.77±0.95	3.73±0.93	4.11±1.04	3.83±0.73
T		2.483*	4.304***	3.397**	2.399*	1.923	5.737***
Inclusive or not	Puhui Garten	3.71±0.86	3.64±0.94	3.80±0.92	3.89±0.89	4.06±1.02	3.82±0.76
	Non-inclusive garten	3.56±1.01	3.48±0.80	3.57±0.99	3.58±0.93	3.46±1.05	3.53±0.67
T		7.910***	5.965***	5.232***	5.899***	6.166***	9.500***
Area	Eastern and coastal	3.67±0.44	3.71±0.31	3.79±0.36	3.69±0.47	3.86±0.35	3.74±0.74
	West	3.41±0.24	3.62±0.29	3.52±0.36	3.40±0.41	3.58±0.27	3.51±0.79
	Central and North	3.34±0.39	3.55±0.32	3.49±0.38	3.13±0.51	3.41±0.33	3.38±0.56
F		3.181*	1.321	0.527	3.740*	4.321*	3.970*

The F-test values of responsibility management, customer responsibility, employee responsibility, and organizational responsibility are 4.812, 2.805, and 2.955, respectively. Still, there is no significant difference between responsibility management and social services. Generally, institutions in the early stage of the establishment have more positive social performance, but the results of this study are different. This may be due to the inadequate ability of institutions in the early stage of the establishment to deal with the complex social environment, risk assessment, and processing capabilities [25]. More experience to improve and accumulate dynamic adaptability and resources may also be affected by the lack of understanding of relevant policies and government subsidies at the initial establishment stage.

F-test values of nursery classes indicate that the larger the values, the better the performance in terms of customer responsibility, employee responsibility, social services, and organization’s responsibility, were 7.151, 4.321, 6.261, 3.396, respectively, with no significant differences in management responsibilities. The results indicate that the resource gap between kindergartens of different sizes is getting larger. The higher the kindergartens’ scale, the more funding sources, and the more opportunities to obtain high-quality educational capital [53]. Therefore, the excessive concentration of resources in smaller kindergartens leads to a scarcity of educational capital. The scarcity of resources makes the institution unable to perform well in terms of social responsibility and inclusive participation, which is consistent with the results of this study.

F-test values of the nature of the nursery show that private kindergartens in liability management, customer responsibility, and employee responsibility were 2.483, 4.304, 3.397, 2.399, and social services performed better than public kindergartens. But, there are no significant differences in terms of organizational responsibility. The analysis reveals that public kindergartens have advantages in resource allocation over private kindergartens but poor educational quality [54]. However, the preschool education market is expanding rapidly, particularly in private kindergartens. The teachers of private kindergartens have more active work dedication and higher levels of work [55]. It is better than public kindergartens in terms of social responsibility and inclusive performance, which is consistent with the results of this study.

F-test values of inclusive or not show that responsibility management, customer responsibility, employee responsibility, and social services were 7.910, 5.965, 5.232, 5.899, and 6.166, respectively. Many scholars’ comprehensive definitions of inclusiveness can be summarized as a universal preference, non-discriminatory, and quality [2,56,57]. An inclusive kindergarten assumes a particular spirit of social service, so it needs to be better in terms of social responsibility performance.

Regarding regional distribution, kindergartens in the eastern and coastal regions (9 regions including Jiangsu, Zhejiang, Shanghai, and Guangdong) performed best in responsibility management, social service, and organizational responsibility aspects, followed by the western region (8 regions including Sichuan, Yunnan, and Chongqing). China’s eastern and coastal areas have developed economies and relatively concentrated resources. Kindergartens have sufficient resources to assume more social responsibilities [58]. They need to actively fulfil their social obligations to survive and develop in the fiercely competitive preschool education market.

## 5. Recommendations for improving the social responsibility of Puhui Garten

To improve the service quality of a kindergarten, it is necessary to fulfil its social responsibilities, permissible education system, the quality evaluation mechanism, the financial sharing mechanism, and the participation model of multiple subjects.

### 5.1 Prioritize policies and strengthen the social responsibility system

The study found that the social responsibility of *Puhui* kindergarten can be performed better when it involves the implementation of the ‘mandatory social responsibility and other laws and regulations. The bottom line fairness concept can promote the public service system. The part ‘below the bottom line’ is guaranteed and rigid and also the responsibility of the government and society. The part ‘above the bottom line’ can be undertaken by enterprises, social organizations, and individuals using flexible market mechanisms [53]. In the construction and operation of the public service system of preschool education, it is necessary to deal with the relationship between rigid and flexible systems. It is needed to give full attention to the government’s inescapable bottom-line responsibility and attach importance to the transfer function of social responsibility and mutual aid charity to strengthen the social responsibility system.

The performance scores are relatively low when it involves the cognitive categories of mobilization, motivation, and promotion under the utilitarian principle of voluntary social responsibility [59]. On the other hand, in the current management system of *Puhui* kindergarten, it is more difficult for parents to understand the situation of their children at school, and even if they know the problem, they have nowhere to give feedback. Therefore, the government should further construct and improve relevant policies and regulations, formulate appropriate and effective social responsibility incentive mechanisms, and follow exposure and punishment measures. It can ensure that *Puhui* kindergartens should be responsible for teaching safety, charging standards, etc.

Taking policies and regulations first, the government should call on and organizes relevant education departments to build a platform for social responsibility assessment of *Puhui* kindergartens. It evaluates the *Puhui* kindergartens with the highest score for fulfilling their social responsibility every year. A certain degree of financial subsidies has also been established as a benchmark in the industry, thereby leading institutions in preschool education can fulfill their social responsibilities [21].

### 5.2 Ensuring quality assurance and establishing an evaluation system

According to the findings, the variation in teaching quality and parent satisfaction in fulfilling the social responsibilities of *Puhui* kindergartens is the most thoughtful. It is urgent to build a social responsibility evaluation index system for *Puhui* kindergartens to effectively encourage and evaluate the construction of the social responsibility system. From the subject of evaluation, kindergarten evaluation can be divided into two aspects: external assessment and self-evaluation. The self-assessment of the kindergarten should emphasize the bottom-up initiative. Kindergarten’s self-assessment involves procedures, content, methods, tools, and skills and should be highly professional. This study found that the resource strength, social service capabilities, and service quality levels of kindergartens in different regions, scales, and stages of development were not uniform. So, it is not easy to complete a practical evaluation work solely based on the kindergarten’s strengths. Only self-assessment and external supervision can ensure preschool education service level [60]. The external evaluation of kindergartens emphasizes the top-down government evaluation. According to the view of new public management, the function of the government is to steer rather than paddle, and government public administration is not ‘regulatory administration’ but ‘service administration’ [61]. The government and kindergartens should have an equal footing and a cooperative relationship in the evaluation process. Accordingly, the government can provide professional evaluation content, methods, and skills training to kindergarten managers and teachers by entrusting experts. It can guide the kindergartens, create good evaluation conditions and environments, and improve their internal and external evaluations.

In addition, the evaluation feedback mechanism between the government and kindergartens must be improved. Evaluation feedback should be a two-way dialogue, negotiation, and exchange process [62]. The government should not only give feedback on the evaluation criteria and results to the kindergarten in time for rectification but also regularly listen to the opinion of the kindergarten managers and teachers on the evaluation. It can help continuous adjustment and optimization of the evaluation mechanism. This practice can make a scientific and democratized kindergarten evaluation and policy system.

### 5.3 Balance resources and improve a reasonable public financial sharing mechanism

The study reveals that kindergartens with different organizational characteristics have various performances in the social responsibility program. The specific manifestation is that the longer the kindergarten has been established, the more classes, the better social responsibility performance. At the same time, the level of social responsibility of private kindergartens is more favorable than that of public kindergartens. It shows that the current public kindergartens have not fully played the role of guaranteeing and balancing the ‘underpinning’ of maintaining educational fairness and justice and the inclusive policy of private kindergartens. At present, the preschool education industry has a suitable environment for implementing inclusive policy. It only needs to optimize the path of resource allocation and explore the path of a blossoming variety of local demands under the top-level design of the central government.

The study also reveals that kindergartens in eastern and coastal areas (such as Jiangsu, Zhejiang, Shanghai, and other eastern and coastal economically developed areas) perform better than other areas. There is a huge difference between the central and local governments’ financial investment in preschool education. The provincial government’s investment is 0.2 times that of the central government, the city government is 1.9 times, the county government is 3.3 times, and the town government’s investment is 20.3 f ^ptimes [63]. It is observed that the current inclusive development of preschool education is mainly through the support and investment of the local government. Economic growth in different regions is uneven, resulting in differences in the importance of grassroots government on preschool education and financial budgets, which is challenging to mobilize the action of inclusive preschool education fully. Accordingly, it is necessary to establish a sound public financial investment sharing mechanism and financial guarantee system and stipulate its principles, content, structure, and ratio. Relevant education administrative departments need to balance the financial investment in preschool education and use special funds to favor small-scale private preschool education institutions. At the early stage of establishment, incentives, evaluation, and other methods help create a good development environment for such kindergartens.

### 5.4 Multi-participation to build a social responsibility co-governance mechanism

Due to multi-stakeholder subjects, there is an urgent need to build *Puhui* kindergartens’ social responsibility cultivation mechanism. It can be led by the government and participate in multiple subjects’ promotion, linkage, and normalized monitoring and supervision. For a long time, the government has had the absolute authority to speak for the development of preschool education, which weakens the participation rights of other external stakeholders. This kind of power imbalance management mechanism needs to seek an intermediary to balance, thus forming a ‘multiple governance’ community.

The government should change the role of manager to cooperator, encourage kindergartens to make full use of community resources, stimulate the awareness of community participation, and actively leverage various social organizations such as industry associations, professional societies, and foundations in ECE. It is necessary to attract all levels of people to build a co-governance mechanism among the government, society, and institutions to develop the social responsibility of the *Puhui* kindergartens [54]. On the other hand, it is also necessary to improve the information disclosure mechanism and enhance the transparency of the services. Educational departments must establish a social responsibility assessment and annual inspection release system at all levels. These can be based on the prior status database platform of kindergarten education and teaching and regularly release the quality of kindergarten education to the society. Social responsibility evaluation results can lead the kindergarten’s external stakeholders to understand basic information such as the basic status of the kindergarten, the quality of education, and the fulfillment of social responsibilities. At the same time, it can provide information support for strengthening the kindergarten’s regular monitoring, quantitative evaluation, and social quality supervision. To build bridges, all stakeholders should participate in the kindergarten quality assurance, social responsibility assessment, etc.

## 6. Conclusion

This paper constructed an inclusive kindergarten social responsibility assessment model, including five dimensions: responsibility management, customer responsibility, employee responsibility, social service, and organizational responsibility. Regarding regional distribution, kindergartens in the eastern and coastal regions performed best in responsibility management, social service, and organizational responsibility, followed by the western region. China’s eastern and coastal areas have developed economies and relatively concentrated resources. The analysis shows that the performance of social responsibility varies with the nature of kindergartens, whether they are inclusive or not, the number of classes, establishment years, and distribution areas. It provides a research foundation for the social responsibility construction of inclusive kindergartens. To improve the quality of *Puhui* kindergartens, it is urgent to build a social responsibility evaluation index system to encourage and evaluate the construction of the social responsibility system. The government should shift from the role of a manager to a cooperator, encouraging kindergartens to make full use of local resources, raising community awareness, and leveraging different social groups in ECE, such as industry associations, professional groups, and foundations.

There are a few limitations to this study. Firstly, the participants from China’s eastern, central, and western regions were not proportionally representative because we focused more closely on the eastern and western differences, which were more representative of China’s development disparity, and the central China sample was thus just a supplement. Second, the indicator system was not subjected to a weighted analysis to assess the relative value of each indication. Third, the findings were mostly reliant on the participants’ subjective opinions.
